# Current gene panels account for nearly all homologous recombination repair-associated multiple-case breast cancer families

**DOI:** 10.1038/s41523-021-00315-8

**Published:** 2021-08-25

**Authors:** Thibaut S. Matis, Nadia Zayed, Bouchra Labraki, Manon de Ladurantaye, Théophane A. Matis, José Camacho Valenzuela, Nancy Hamel, Adrienne Atayan, Barbara Rivera, Yuval Tabach, Patricia N. Tonin, Alexandre Orthwein, Anne-Marie Mes-Masson, Zaki El Haffaf, William D. Foulkes, Paz Polak

**Affiliations:** 1 Department of Human Genetics, McGill University, Montreal, QC Canada; 2 Cancer Research Program, Centre for Translational Biology, The Research Institute of the McGill University Health Centre, Montreal, QC Canada; 3 Cancer Genetics Unit, Institut Bergonié, Bordeaux, France; 4 Divison de Médecine Génétique, CRCHUM, Montreal, QC Canada; 5 Centre de recherche du Centre hospitalier de l’Université de Montréal and Institut du cancer de Montréal, Montreal, Quebec Canada; 6 Ecole Supérieur de Génie Informatique, Paris, France; 7 Gerald Bronfman Department of Oncology, McGill University, Montreal, QC Canada; 8 Lady Davis Institute for Medical Research, Jewish General Hospital, Montreal, QC Canada; 9 IDIBELL Bellvitge Biomedical Research Institute, Barcelona, Spain; 10 Department of Developmental Biology and Cancer Research, Institute for Medical Research-Israel-Canada, Hebrew University of Jerusalem, Jerusalem, Israel; 11 Department of Medicine, McGill University, Montréal, QC Canada; 12 Department of Medicine, Université de Montréal, Montreal, QC Canada; 13 Department of Oncological Sciences, Icahn School of Medicine at Mount Sinai, New York, NY USA

**Keywords:** Breast cancer, Genetics research

## Abstract

It was hypothesized that variants in underexplored homologous recombination repair (HR) genes could explain unsolved multiple-case breast cancer (BC) families. We investigated HR deficiency (HRD)-associated mutational signatures and second hits in tumor DNA from familial BC cases. No candidates genes were associated with HRD in 38 probands previously tested negative with gene panels. We conclude it is unlikely that unknown HRD-associated genes explain a large fraction of unsolved familial BC.

Many multiple-case BC families remain unexplained by a pathogenic variant, despite routine clinical testing of an increasing number of recognized BC susceptibility genes (BCSGs). These cases pose a major clinical challenge for disease management of patients and cancer prevention.

To identify germline pathogenic variants (GPVs) in patients, Next-Generation Sequencing (NGS) of blood-derived DNA is used, often through pan-cancer panels containing up to 25 genes. The three major BCSGs – BRCA1, BRCA2, and PALB2 – are involved in homology-directed DNA repair (HR). Several other, less well-studied genes, also involved in DNA repair and all variably associated with increased risk for hereditary breast and ovarian cancer (HBOC), are also included on some panels. As PARP inhibitors have been approved for treating cancers with HR defects, it is important to identify all possible HR pathway genes reliably implicated in BC susceptibility. To this end, many clinical BC susceptibility genes (BCSGs) testing panels include putative BC-related HR pathway genes, but their candidacy remains unproven. This can lead to inappropriate use of expensive therapies.

We and others showed that tumor sequencing is a powerful tool to find genes or variants that cause HRD breast cancer. In BC cases due to known BCSGs, the inherited pathogenic variant confers genetic susceptibility, but tumorigenesis is usually driven by a somatic inactivating “second-hit” event resulting in loss of gene function, often via loss of heterozygosity (LOH) through deletion of the wild-type allele. In this tumor suppressor model of BCSG function, as a nascent tumor cell loses the ability to repair DNA, lesions accumulate, including in genes favoring tumor progression^1^. Tumor genomes of patients with *BRCA1/2* inactivation were found to be enriched in copy number losses and rearrangements. This observation led to the development of an HRD index based on these somatic genomic abnormalities that can be used to detect HRD implicated tumors rather than on the specific mutational spectrum at the nucleotide level of the altered the DNA sequence^2–5^. In addition, in tumors with anomalies in HR repair, single base-pair changes have been shown to accumulate in the tumor genome, giving rise to what is referred to as mutational signatures. We showed that using sequencing of paired tumor and normal tissues we can detect these HRD-associated mutational signatures as well as second hits^6,7^. COSMIC tumor mutational signature 3 (Sig 3) has been associated with homologous repair deficiency (HRD)^8^. Monoallelic inactivation of *BRCA1/2* does not give rise to Sig 3, whereas Sig 3 is seen in tumors with biallelically inactivated *BRCA1/2*.

The mutational signature analysis helped to define the role of various HR genes directly from case-only data by association with Sig 3 levels. We showed that inactivation of *RAD51C*, *RAD51D*, *PALB2*, and *BARD1* are all associated with elevated Sig 3 levels, indicating that GPVs in these genes lead to *BRCA*-like/HRD cancers. In contrast, GPVs in other established and candidate BCSG genes such as *ATM*, *ATR*, *BRIP1, MRE11*, *NBN*, and *RAD50* do not lead to BRCA-like tumors^6^. Moreover, we developed a framework to reclassify germline *BRCA1/2* variants of unknown significance (VUS) as benign or pathogenic variants in the context of HRD in tumors^6^. A variant was classified as a pathogenic or disease-causing variant if the tumor that harbored it showed a second hit and Sig 3. This approach had 100% sensitivity and 98.5 % specificity in classifying 142 known benign and pathogenic missense mutations in *BRCA1/2*. Thus, Sig 3 levels can be used to identify new HR genes and new pathogenic variants in genes that lead to BRCA-like tumors.

In this study, we designed a pipeline encompassing variant calling and detection of second events involving somatic variants, combined with mutational signature analysis for HRD detection. The pipeline was optimized for archival formalin-fixed paraffin-embedded (FFPE) tumor tissues. We studied 38 unsolved BC patients with either early-onset BC or a strong family history of BC with no identified GPVs in known BCSGs (Supplementary Table 1). We tested the hypothesis that their tumors are linked to HRD due to alterations in genes that have not yet been definitively found associated with heritable risk to BC (Fig. 1).

**Fig. 1:**
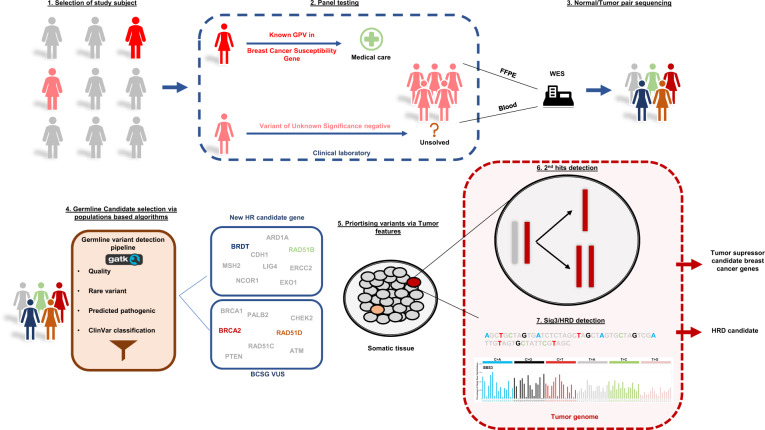
Study workflow. (1) all eligible women were selected based on their personal and family history of cancer (red and pink women) and (2) received prior clinical screening. (3) unsolved cases were selected for WES through normal/tumor pair samples from blood lymphocytes and FFPE tissues. (4) sequencing data were analyzed through a germline candidate variant detection algorithm focused on homology-directed repair candidates following a classical BCSG model. (5) tumor landscapes were evaluated through a variant calling pipeline designed to minimize artifacts and a second-hit detection strategy based on the biological mechanism (i.e. defects in the hr pathway). (6) candidate BCSGs were re-evaluated in the context of a patient harboring a germline variant with an HRD tumor and second hit.

To identify possible genetic causes that underly BC in our cases, we compiled a list of candidate genes implicated in HR which are not on current genetic testing panels (see methods). We analyzed whole-exome sequencing data (WES) of tumor/normal pairs to look for potentially pathogenic rare variants and to search for evidence of a second hit in the tumor based on a tumor suppressor gene model as well as for evidence of an HRD signature^6^ (Fig. 1).

Three identified variants were considered as pathogenic by ClinVar: two variants were in the *FANCA* and *GJB2* genes, but neither showed a second hit. The third was a nonsense variant in *LIG4* that showed LOH in the tumor. We observed LOH of the WT allele for 18 additional germline variants. We found five VUSs and three variants with Conflicting Interpretation of pathogenicity (CIOP) in *APC, ATM*, *BRCA2*, *CHEK2*, *FLCN*, and *RAD51D*; and the remaining variants were not classified in ClinVar (Fig. 2, Supplementary Fig. 1). No second somatic LOF alteration was found in combination with a germline candidate variant. All variants are summarized in Tables 1, 2.

**Fig. 2:**
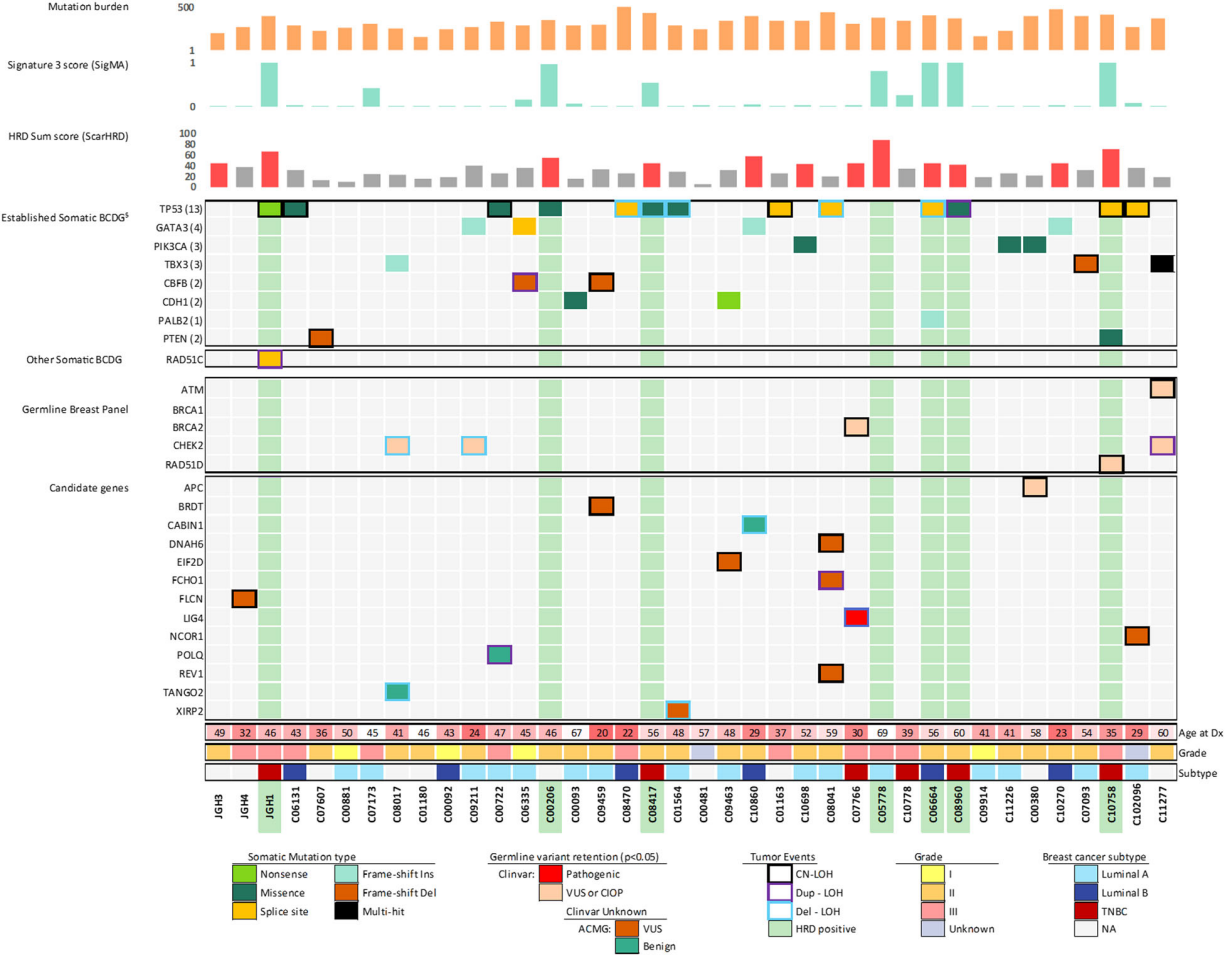
Co-mutation plot of germline and somatic landscape after HRD detection. The HRD status evaluated by SigMA (that estimates the presence of Signature 3) is shown in the second row from the top, and columns with HRD based on SigMA are highlighted in vertical green columns. ScarHRD, shown in row 3, calculates the HRD index and cases with HRD determined by this method that has scores great than 41 are represented by red bars. The germline and somatic mutation status of candidate genes across patients are shown for four classes of genes (rows 4–7). BCDG: Breast Cancer Driver Gene; CN-LOH: copy neutral LOH; Dup-LOH: Duplication with LOH; Del-LOH: hemizygous deletion with LOH.

**Table 1:** List of missenses variants retained in the tumor by LOH.

Gene	Patient ID	NM number	DNA changes	Amino Acid changes	Variant consequences	Gene category	ClinVar classification	ACMG classification	Variant retention type	Germline REF read counts	Germline ALT read counts	Tumor REF read counts	Tumor ALT read counts	*p* value
APC	C00380	NM_001127510	c.8275C>T	p.R2759C	Missense	CSG	VUS	–	CN-LOH	80	64	134	177	0.013197
ATM	C11277	NM_000051	c.1538A>G	p.Q513R	Missense	BCSG	VUS	–	CN-LOH	191	142	8	24	0.000446
BRCA2	C07766	NM_000059	c.9744G>A	p.M3248I	Missense	BCSG	VUS	–	CN-LOH	114	97	13	52	0.000669
BRDT	C09459	NM_001242805	c.272C>T	p.A91V	Missense	C-HRG	Unknown	VUS	CN-LOH	42	35	12	38	0.00129
CABIN1	C10860	NM_012295	c.2446C>T	p.L816F	Missense	C-HRG	Unknown	Likely benign	DEL-LOH	112	107	32	55	0.032096
CHEK2	C08017	NM_001005735	c.628G>A	p.G210R	Missense	BCSG	CIOP	–	DEL-LOH	211	155	22	103	<0.00001
CHEK2	C09211	NM_001005735	c.1165C>T	p.R389C	Missense	BCSG	VUS	–	DEL-LOH	114	128	36	67	0.037132
CHEK2	C11277	NM_001005735	c.1465A>G	p.N489D	Missense	BCSG	VUS	–	DUP-LOH	192	180	23	49	0.002237
EIF2D	C09463	NM_006893	c.1538A>G	p.Y513C	Missense	C-HRG	Unknown	VUS	CN-LOH	35	36	136	406	0.000016
FCHO1	C07766	NM_001161357	c.557G>A	p.R186Q	Missense	C-HRG	Unknown	VUS	DUP-LOH	155	148	31	87	<0.00001
FLCN	JGH4	NM_144997	c.715C>T	p.R239C	Missense	CSG	CIOP	–	CN-LOH	109	120	44	185	<0.00001
NCOR1	C102096	NM_006311	c.2794C>T	p.H932Y	Missense	C-HRG	Unknown	VUS	CN-LOH	138	101	34	99	<0.00001
POLQ	C00722	NM_199420	c.1981T>G	p.W661G	Missense	C-HRG	Unknown	Likely Benign	DUP-LOH	26	28	7	21	0.042667
RAD51D	C10758	NM_002878	c.620C>T	p.S207L	Missense	BCSG	CIOP	–	CN-LOH	129	120	13	132	<0.00001
REV1	C08041	NM_001321460	c.2017G>A	p.D673N	Missense	DNA Repair Gene	Unknown	VUS	CN-LOH	88	80	60	93	0.01811

For the three CIOP variants, *FLCN* c.715C>T is trending towards benign https://www.ncbi.nlm.nih.gov/clinvar/variation/134427/ and is not a plausible BCSG. *RAD51D* c.620 C>T is discussed above and is recorded as likely pathogenic by most laboratories https://www.ncbi.nlm.nih.gov/clinvar/variation/142102/. *CHEK2* c.628 G>A (also known as NM_007194.4(CHEK2):c.499 G>A (p.Gly167Arg)) is reported as likely pathogenic by nearly all laboratories on ClinVar https://www.ncbi.nlm.nih.gov/clinvar/variation/142524/ and has been reported to result in a severe cancer phenotype in the homozygous state^40^. While the presence of LOH in the case reported here could contribute to its final classification on ClinVar as Pathogenic, biallelic *CHEK2* mutations are not associated with any known mutational signature 3 and therefore our approach cannot be used to reclassify this or any other *CHEK2* variant.

*BCSG* breast cancer susceptibility genes, *CSG* cancer susceptibility genes, *C-HRG* candidate homologous recombination genes, *DEL-LOH* LOH by deletion of the WT allele, *DUP-LOH* LOH by multiplication of the alternate variant and loss of the WT allele, *CN-LOH* LOH by copy neutral event, *VUS* variant of uncertain significance, *CIOP* variant with conflicting interpretations of pathogenicity.

**Table 2:** List of truncating variants retained in the tumor by LOH.

Gene	Patient ID	NM number	DNA changes	Amino Acid changes	Variant consequences	Frequency of LoF	Gene category	ClinVar classification	ACMG classification	Variant retention type	Germline REF read counts	Germline ALT read counts	Tumor REF read counts	Tumor ALT read counts	p value
DNAH6	C08041	NM_001370	c.6184delG	p.D2062Mfs*19	frameshift del	1.08%	C-HRG	Unknown	Likely Pathogenic	DEL-LOH	79	34	35	48	0.000181
LIG4	C07766	NM_206937	c.2440C>T	p.R814X	nonsense	0.16%	DNA Repair Gene	Pathogenic	-	DEL-LOH	148	83	29	40	0.001089
TANGO2	C08017	NM_001322149	c.158C>G	p.S53X	nonsense	1.22%	C-HRG	Unknown	Benign	CN-LOH	194	187	49	204	<0.00001
XIRP2	C01564	NM_152381	c.8352delG	p.L2784Ffs*43	frameshift del	1.44%	C-HRG	Unknown	Likely Pathogenic	DEL-LOH	69	56	18	33	0.016566

LoF frequencies (LF) for an *x* gene were calculated through LF(*x*) = $2 \times \left( {\frac{{S\left( x \right)}}{{m(x)}}} \right)$ where *S* is the allelic count of LoF variant, *m* the median of all allele number in gnomAD v2.1.1.

To identify patients with HRD-related tumors, we used two HRD classifiers tools. SigMA is tailored for WES tumor data and is used to identify Sig 3 (Fig. 2, Supplementary Fig. 2). We also used ScarHRD, which is based on evaluating genomics scars due to HRD in tumor DNA and has been recently adapted for use with WES data. We identified 7 HRD tumors based on SigMA, and 12 based on ScarHRD (Supplementary Fig. 3). All 7 cases exhibiting an HRD signature by SigMA analysis were those also classified as having genomic scars consistent with HR defects by ScarHRD. Given that WES is not ideally suited to detect large genomic events, we considered the 7 tumors (18%) that were called positive by both tools for further analysis (Fig. 2). This percentage is within the range of detection reported by other HRD studies^7,9–11^. The only HRD tumor with a germline BCSG candidate variant was from a BC patient with the missense variant *RAD51D* p.S207L (case C10758). Using copy number analysis, we were able to identify duplication of the germline missense variant along with a *trans* allele loss, resulting in copy neutral LOH (CN-LOH) event at the gene locus as the second hit. We found a somatic deletion at a donor splice-site in *RAD51C* associated with duplication-LOH in another tumor with HRD (case JGH1), without germline events (Fig. 2). No other HRD tumor was found to be associated with our candidate genes either somatically or in the germline.

Variants in known BC drivers^12^ were found in 84% of tumors (Fig. 2, Supplementary Fig. 4). Of these, *TP53, GATA3, and CBFB* were considered as a driver (see methods). Three patients harbored a *PIK3CA* hotspot mutation. We also detected somatic alterations in three BC genes (*PTEN, PALB2*, and *CDH1*) in 4 other patients. Copy number profiles were similar to observations from previous BC cohort studies^12,13^.

Our pipeline worked well in calling variants in archival FFPE tumors, which are more accessible than fresh tumor material. Except for the *RAD51D* variant, which has a conflicting interpretation of pathogenicity (https://www.ncbi.nlm.nih.gov/clinvar/variation/142102/), we did not find supporting evidence to reclassify any ClinVar-described variants found in our HRD tumors as pathogenic. The fact that we were able to independently identify p.S207L *RAD51D* variant as a candidate pathogenic variant using our pipeline demonstrates the robustness of the genomic tools used in this study to reassess VUS. Although we sequenced a highly selected population of BC patients from multiple-case BC families, we did not find any other plausible GPVs in genes beyond those currently included in the clinical gene testing panels, which is in line with recent studies^14^. We focused on HRD as a causal mechanism in BC, but our approach could be of use to identify germline drivers in other tumor types with mutational signatures that are associated with defects in DNA repair pathways, such as mismatch repair, hyper-mutated tumors, and base excision repair.

There are a number of caveats to our study. Our inability to detect potentially pathogenic variants in recently confirmed BCSGs (e.g. *RAD51C* and *BARD1*^10,15^) as well in largely unexplored HR genes could be due to the small sample size of our study. As WES is not ideally suited to identify large structural rearrangements associated with HR defects we could have missed tumor harboring anomalies due to HRD. Also, it is possible that undiscovered BCSGs do not operate via the HR pathway, and in this case our HRD-based framework will not detect variants in these genes. Finally, we cannot exclude the possibility that variants were missed for technical reasons given the age of some of the FFPE material, as one-quarter of our cases were diagnosed more than 10 years prior to analysis, which likely affected the quality of DNA for analysis. This limitation would not be as relevant for somatic testing of freshly processed tissues in a clinical context. Importantly, our study focused on WES data, so we could not assess other molecular mechanisms affecting gene function as potential contributors, such as complex structural variations^9^ or somatic methylation events^16^.

Nones et al.^17^ took a similar approach using fresh frozen tumors and were able to show that all but one causally unexplained BCs with HRD were due to inactivation of *BRCA1*, *BRCA2*, or *PALB2*. Taken together with the results we present here, we can conclude that the tail of missing heritability due to HRD genes must be long and thin. It also means that existing gene testing panels are sufficient to identify carriers of clinically important BC genes using a normal/tumor pair sequencing-based approach. We must therefore look elsewhere to find the missing inherited susceptibility present in these families.

## Methods

### Samples selection

This study called “Genome-wide approaches in hereditary cancer families”, study number A08-M61-09B, was created in 2011 and approved by the McGill University Research Ethics Board. Participants to this study were consented at two Montreal sites. Some participants were consented directly into the scientific study at McGill University and its affiliated hospitals. Others were recruited from a Montreal-based biobank entitled “Banque d'échantillons biologiques et de données (cliniques et biologiques) associées à des fins de recherche sur les cancers métastatiques du sein et de l’ovaire” previously created in 2000 and approved by the CHUM Institutional Review Board, approval number BD 04.002. We identified over 70 candidate families eligible for this study. All had early-onset BC or a strong family history of breast cancer (median Manchester score 24, range 10-59). None carried a *BRCA1/2* or *PALB2* pathogenic or likely pathogenic variant on clinical genetic testing. We reviewed approximately 130 pathology blocks to retrieve available BC tumor material that would be suitable for analysis. Blood samples were only obtained from patients where tumor material was successfully retrieved and processed. Of 59 FFPE tumors with extracted DNA, 20 samples failed sequencing due either to poor quality or insufficient quantity of DNA. WES was thus performed on 39 normal/tumor pairs of women with BC diagnosed between 1995 and 2017, but we removed one because of unresolved contamination issues. The interval between diagnosis and sequencing ranged from a few months to 22 years (median 3 years).

### Normal/tumor WES sequencing

Genomic DNA was extracted from the patient’s peripheral blood lymphocytes and tumor using published commercial protocols and kits optimized in our lab (Maxwell RSC Buffy Coat DNA/Tissue DNA kits, Promega). Library preparation, exome capture, and sequencing were conducted over a period of 2 years, as material was obtained, reflecting repeat assays for DNA samples that failed. A Panel of Normals (PoN) comprised of 47 normal tissue samples, all sequenced using the same protocol as we applied to tumor tissues, was used to assist in removing recurrent technical artifacts due to formalin fixation. Genomic DNA libraries were generated using one of the following: Nextera Rapid Capture Exome Target (Illumina, San Diego, California), Sure Select Human All Exon V6, or Sure Select Human All Exon V7 (Agilent). DNA samples were then sequenced using Illumina sequencers and appropriate protocols available at the time of sequencing: Illumina HiSeq 2000 as 100 bp paired-end reads, Illumina HiSeq 2500 as 125 bp paired-end reads, and Illumina HiSeq 4000 as 100 bp paired-end reads (Illumina, San Diego, California).

### Germline variant calling and filtering

Exome sequencing data analysis was performed using the pipeline we developed for this project. Briefly, BWA (v. 0.5.9) and the Genome Analysis Toolkit (GATK)^18^ were used to align the sequenced reads to the reference genome (UCSC hg19) and to perform local realignment of reads around small insertions and deletions (indels). PCR duplicate reads were marked using Picard (https://github.com/broadinstitute/picard). The coverage of consensus coding sequence (CCDS) bases was calculated by GATK, resulting in a mean coverage of 141.3X (ranging from 26.9X to 250.6X) and 97.3% of CCDS bases were covered by at least 10 reads. Germline variants were called individually for each sample using HaplotypeCaller from GATK and annotated by wAnnovar^19^. From the generated list of germline variants, variants were filtered out if they fulfilled any one of the following quality criteria: (i) genomic position of variant covered by <5 reads; (ii) <2 reads support the alternative variant; (iii); allelic fraction between < 0.35 or >0.5; (iv) and mapping quality < 30. We then focused on four categories of genes (n = 864): 20 BCSG frequently used in clinical testing, and previously tested in our HBC cases; 594 “Candidate HR genes” list (C-HRGs) purported to have co-evolved^20^ or known to interact in HR (unpublished data from Dr. Alexander Orthwein); 103 known DNA repair genes; and 147 other known Cancer Susceptibility Genes (CSGs)^21^ (Supplementary Table 2). Only variants having an allele frequency <0.01 based on allele frequencies in GnomAD^22^ and 1000 Genomes project^23^ were retained for further investigation.

### Somatic variant calling and filtering

Somatic variants were called individually through normal/tumor paired caller using Mutect2^24^ for SNV and indel. The PoN was used as well as germline population resources according to GATK’s best practice. Variants were filtered out if they (i) displayed strand bias, to remove artifacts caused by the formalin fixation process; (ii) were germline in origin; (iii) were due to technical artifacts revealed by comparison to PoN, and (iv) had a depth < 10 reads. Variants were next annotated with VEP^25^.

### Somatic Copy Number Analysis and LOH detection

With GATK CNA, read coverage counts across the target were collected. We created a separate copy number variation (CNV) PoN to reduce read coverage data noise to obtain denoising copy ratios against the PoN. Then we collected counts of reference versus alternate alleles for allelic imbalance. We used GISTIC^26^ to assess recurrent focal amplifications and deletions in tumors. We used FACETS^27^ and Sequenza^28^ to infer the integer copy number of each pair of alleles as well as LOH. Hemizygous deletions were defined as the loss of one allele in the tumor. Homozygous deletions were defined as the loss of both alleles in the tumor. Amplification was defined as a copy number status ≥ 6 per tumor for *ERBB2*, *MYC*, and *ZNF703*.

### Variant visualization

The Integrative Genomics Viewer^29^ was used for the manual examination and visualization of all germline candidate variants, and somatic events.

### Somatic cancer driver gene identification

A combination of several tools was used to infer drivers of oncogenesis based on statistical methods through q-score to identify genes that were mutated more often than expected by chance: MutSigCV^30^, OncodriveFML^31^, MutPanning^32^, and FishHook^33^. Genes that were found mutated more than once with a q-score < 0.05 were considered as cancer driver genes.

### Mutational signature and homologous recombination repair deficiency detection

Different tools were used to assess the mutational signature from normal-tumor paired WES VCF data: MutationalPatterns^34^, Maftools^35^, DeconstructSig^36^ for tumor signature clustering and the mutational signature contribution in each tumor. Different approaches were used: a de novo mutational signature extraction using a non-negative matrix factorization (NMF) and a COSMIC signature similarity using cosine similarity. We used SigMA^37^ to detect the mutational signature associated with HR defect designed for WES. We also used ScarHRD^38^ to detect HRD genomic scars—genomic abnormalities characteristic of HRD, which is based on the sum of LOH score, Large Scale Transition (LST) score, and Telomeric Allelic Imbalance (TAI) score. A score of ≥ 42 classifies tumors as HRD (see Supplementary Fig. 3).

### Candidate variant selection algorithm

A list of candidate pathogenic variants was generated using our variant detection algorithm (Supplementary Fig. 5). ClinVar annotations were used to classify variants already reported, and when no ClinVar data was available, we used the ACMG criteria. Variants classified as likely benign or benign by ClinVar were filtered out. The master list of variants was comprised of truncating, missense, or splice-site alterations found in gene candidates.

For non BSCG, missense and splice-site variants predicted to be deleterious by at least 4 of 8 bioinformatic prediction tools (SIFT, PolyPhen2, MutationTaster, FATHM, Provean, MetaSVM, MetaLR, and CADD) were considered for further analysis. For known BSCG genes, we considered all variants regardless of scores as these can be evaluated by expertise. Truncating variants were classified separately. The candidate list was comprised of 231 variants: 7 missense VUSs or variants with conflicting interpretations of pathogenicity in known BCSGs, 46 truncating variants, and 178 predicted pathogenic variants in C-HRGs, DNA repair genes, and in CSGs. From this list of candidate genes, we searched for evidence of allelic imbalance at each gene locus in the tumor using statistical tests (Chi-squared test) and filtered out variants where the p-value was >0.05 and confirmed with FACET results that there was a deletion of one haplotype (see above). We also looked for somatic second-hit point mutations. Our final list of candidate variants was comprised of genes that showed evidence of a possible second mutational hit in the tumor.

### Reporting summary

Further information on research design is available in the Nature Research Reporting Summary linked to this article.

## Supplementary information

Supplementary Figures

Dataset 1

Reporting Summary

## Data Availability

The data generated and analyzed during this study are described in the following data record: 10.6084/m9.figshare.14802954^39^. Tumor and germline datasets generated during the current study are not publicly available as the consent provided by study participants did not include a provision for widespread disclosure. However, direct requests can be made to the corresponding authors for access. The following files are openly available as part of the figshare data record: ‘Master_list_variant.xlsx’ (underlying Table 1), ‘Genes assessed initially.xlsx’ (underlying Supplementary Table 1), ‘Candidate Genes list.xlsx’ (underlying Supplementary Table 2). All other data are housed on institutional storage and are not openly available in order to protect patient privacy as informed consent to share participant-level data was not obtained prior to or during data collection. However, these data can be requested from Dr Foulkes. The files are: ‘all germline_sample.vcf’, ‘data.tumor.maf’, ‘all tumor_sample.vcf’, ‘all sample.out.gzsmall.seqz.gz’.

## Code availability

The bioinformatics analyses were performed using open-source software, including Burrows–Wheeler alignment tool (v. 0.5.9), GATK (v.4.1.6.0), samtools (v.1.10), bcftools (v.1.10), htslib (v.1.10), Picard (v.2.24.2), vcf2maf (v.1.6.17), R (v.4.0.3), CNV_facets (v.0.16.0), Maftools (v.0.9.30), FishHook (v.1.0), Bioconductor (v.3.12), MutationalPatterns (v.3.0.1), deconstructSigs (v.1.0), SigMA (v.1.0), Sequenza (v.3.0.0) and ScarHRD (v.1.0).

## References

[1] Roy R, Chun J, Powell SN (2011). BRCA1 and BRCA2: different roles in a common pathway of genome protection. Nat. Rev. Cancer.

[2] Abkevich V (2012). Patterns of genomic loss of heterozygosity predict homologous recombination repair defects in epithelial ovarian cancer. Br. J. Cancer.

[3] Popova T (2012). Ploidy and large-scale genomic instability consistently identify basal-like breast carcinomas with BRCA1/2 inactivation. Cancer Res..

[4] Birkbak NJ (2012). Telomeric allelic imbalance indicates defective DNA repair and sensitivity to DNA-damaging agents. Cancer Discov..

[5] Telli ML (2016). Homologous recombination deficiency (HRD) score predicts response to platinum-containing neoadjuvant chemotherapy in patients with triple-negative breast cancer. Clin. Cancer Res..

[6] Polak P (2017). A mutational signature reveals alterations underlying deficient homologous recombination repair in breast cancer. Nat. Genet..

[7] Davies H (2017). HRDetect is a predictor of BRCA1 and BRCA2 deficiency based on mutational-signatures. Nat. Med..

[8] Nik-Zainal S (2012). Mutational processes molding the genomes of 21 breast cancers. Cell.

[9] Nguyen L, W. M. Martens J, Van Hoeck A, Cuppen E (2020). Pan-cancer landscape of homologous recombination deficiency. Nat. Commun..

[10] Riaz N (2017). Pan-cancer analysis of bi-allelic alterations in homologous recombination DNA repair genes. Nat. Commun..

[11] Wang YK (2017). Genomic consequences of aberrant DNA repair mechanisms stratify ovarian cancer histotypes. Nat. Genet..

[12] Nik-Zainal S (2016). Landscape of somatic mutations in 560 breast cancer whole-genome sequences. Nature.

[13] Sircoulomb F (2011). ZNF703 gene amplification at 8p12 specifies luminal B breast cancer. EMBO Mol. Med..

[14] Narod SA (2021). Which genes for hereditary breast cancer?. N. Engl. J. Med..

[15] Breast Cancer Association Consortium. (2021). Breast cancer risk genes—association analysis in more than 113,000 women. N. Engl. J. Med..

[16] Brianese RC (2018). BRCA1 deficiency is a recurrent event in early-onset triple-negative breast cancer: a comprehensive analysis of germline mutations and somatic promoter methylation. Breast Cancer Res. Treat..

[17] Nones K (2019). Whole-genome sequencing reveals clinically relevant insights into the aetiology of familial breast cancers. Ann. Oncol..

[18] McKenna A (2010). The Genome Analysis Toolkit: a MapReduce framework for analyzing next-generation DNA sequencing data. Genome Res..

[19] Chang X, Wang K (2012). wANNOVAR: annotating genetic variants for personal genomes via the web. J. Med. Genet..

[20] Sherill-Rofe D (2019). Mapping global and local coevolution across 600 species to identify novel homologous recombination repair genes. Genome Res..

[21] Huang K (2018). Pathogenic germline variants in 10,389 adult cancers. Cell.

[22] Karczewski KJ (2020). The mutational constraint spectrum quantified from variation in 141,456 humans. Nature.

[23] The 1000 Genomes Project Consortium. (2015). A global reference for human genetic variation. Nature.

[24] Cibulskis K (2013). Sensitive detection of somatic point mutations in impure and heterogeneous cancer samples. Nat. Biotechnol..

[25] McLaren W (2016). The ensembl variant effect predictor. Genome Biol..

[26] Mermel CH (2011). GISTIC2.0 facilitates sensitive and confident localization of the targets of focal somatic copy-number alteration in human cancers. Genome Biol..

[27] Shen R, Seshan VE (2016). FACETS: allele-specific copy number and clonal heterogeneity analysis tool for high-throughput DNA sequencing. Nucleic Acids Res..

[28] Favero F (2015). Sequenza: allele-specific copy number and mutation profiles from tumor sequencing data. Ann. Oncol..

[29] Thorvaldsdóttir H, Robinson JT, Mesirov JP (2013). Integrative Genomics Viewer (IGV): high-performance genomics data visualization and exploration. Brief. Bioinform..

[30] Lawrence MS (2013). Mutational heterogeneity in cancer and the search for new cancer-associated genes. Nature.

[31] Mularoni L, Sabarinathan R, Deu-Pons J, Gonzalez-Perez A, López-Bigas N (2016). OncodriveFML: a general framework to identify coding and non-coding regions with cancer driver mutations. Genome Biol..

[32] Dietlein F (2020). Identification of cancer driver genes based on nucleotide context. Nat. Genet..

[33] Imielinski M, Guo G, Meyerson M (2017). Insertions and deletions target lineage-defining genes in human cancers. Cell.

[34] Blokzijl F, Janssen R, van Boxtel R, Cuppen E (2018). MutationalPatterns: comprehensive genome-wide analysis of mutational processes. Genome Med..

[35] Mayakonda A, Lin D-C, Assenov Y, Plass C, Koeffler HP (2018). Maftools: efficient and comprehensive analysis of somatic variants in cancer. Genome Res..

[36] Rosenthal R, McGranahan N, Herrero J, Taylor BS, Swanton C (2016). DeconstructSigs: delineating mutational processes in single tumors distinguishes DNA repair deficiencies and patterns of carcinoma evolution. Genome Biol..

[37] Gulhan DC, Lee JJ-K, Melloni GEM, Cortés-Ciriano I, Park PJ (2019). Detecting the mutational signature of homologous recombination deficiency in clinical samples. Nat. Genet..

[38] Sztupinszki Z (2018). Migrating the SNP array-based homologous recombination deficiency measures to next generation sequencing data of breast cancer. NPJ Breast Cancer.

[39] Matis, T. S. et al. Metadata record for the article: Current gene panels account for nearly all homologous recombination repair-associated multiple-case breast cancer families. figshare 10.6084/m9.figshare.14802954 (2021). 10.1038/s41523-021-00315-8 PMC8387362 34433815

[40] Paperna T (2020). Homozygosity for CHEK2 p.Gly167Arg leads to a unique cancer syndrome with multiple complex chromosomal translocations in peripheral blood karyotype. J. Med. Genet..

