# Supplementary material for: Current gene panels account for nearly all homologous recombination repair-associated multiple-case breast cancer families
Source: NPJ Breast Cancer. 2021 Aug 25;7:109. doi: 10.1038/s41523-021-00315-8 (PMC8387362; doi:10.1038/s41523-021-00315-8)
Supplement: Supplementary file 1 — Supplementary Figures [file 41523_2021_315_MOESM1_ESM.pdf]

Supplementary Tables:

<u>1). Genes assessed initially</u>. Here is listed all genes previously tested in clinic for each patient. Tests came from different commercial panels.

<u>2). Candidate Genes list.</u> Aside from Breast Cancer Predisposition Gene, we classified our candidate genes into three categories: genes being a part of DNA repair genes, genes involved in all cancer predisposition syndrome, and genes that seem to being involved or interacting with homologous recombination pathway.

Supplementary Figures:

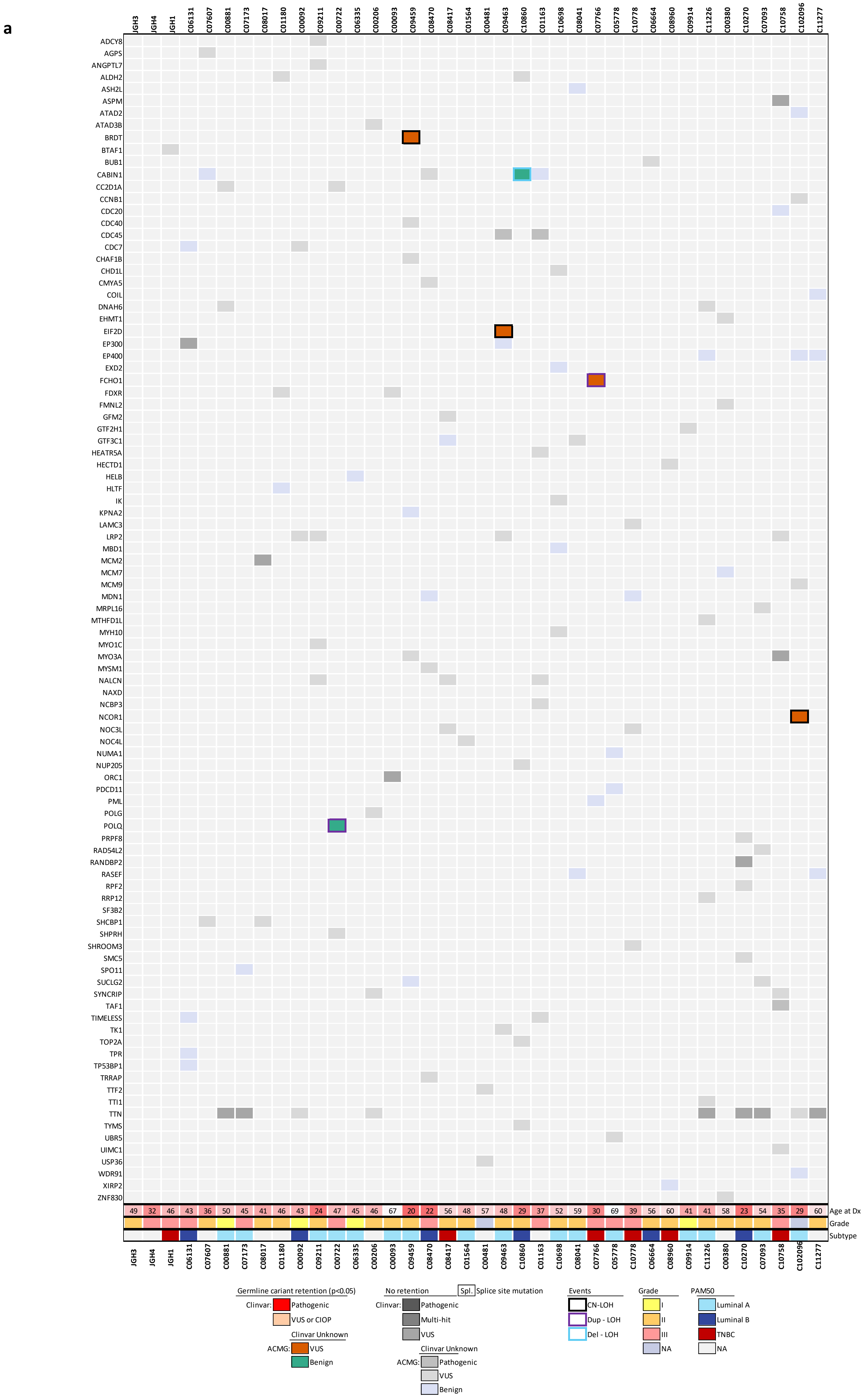

a
JGH3
JGH4
JGH1
C06131
C07607
C00881
C07173
C08017
C01180
C00092
C09211
C00722
C06335
C00206
C00093
C09459
C08470
C08417
C01564
C00481
C09463
C10860
C01163
C10698
C08041
C07766
C05778
C10778
C06664
C08960
C09914
C11226
C00380
C10270
C07093
C10758
C102096
C11277
ADCY8
AGPS
ANGPTL7
ALDH2
ASH2L
ASPM
ATAD2
ATAD3B
BRDT
BTAF1
BUB1
CABIN1
CC2D1A
CCNB1
CDC20
CDC40
CDC45
CDC7
CHAF1B
CHD1L
CMYA5
COIL
DNAH6
EHMT1
EIF2D
EP300
EP400
EXD2
FCHO1
FDXR
FMNL2
GFM2
GTF2H1
GTF3C1
HEATR5A
HECTD1
HELB
HLTF
IK
KPNA2
LAMC3
LRP2
MBD1
MCM2
MCM7
MCM9
MDN1
MRPL16
MTHFD1L
MYH10
MYO1C
MYO3A
MYSM1
NALCN
NAXD
NCBP3
NCOR1
NOC3L
NOC4L
NUMA1
NUP205
ORC1
PDCD11
PML
POLG
POLQ
PRPF8
RAD54L2
RANDBP2
RASEF
RPF2
RRP12
SF3B2
SHCBP1
SHPRH
SHROOM3
SMC5
SPO11
SUCLG2
SYNCRIP
TAF1
TIMELESS
TK1
TOP2A
TPR
TP53BP1
TRRAP
TTF2
TTI1
TTN
TYMS
UBR5
UIMC1
USP36
WDR91
XIRP2
ZNF830
49 32 46 43 36 50 45 41 46 43 24 47 45 46 67 20 22 56 48 57 48 29 37 52 59 30 69 39 56 60 41 41 58 23 54 35 29 60
Age at Dx
Grade
Subtype
Germline cariant retention (p<0.05)
Clinvar:
Pathogenic
VUS or CIOP
Clinvar Unknown
ACMG:
VUS
Benign
No retention
Clinvar:
Pathogenic
Multi-hit
VUS
Clinvar Unknown
ACMG:
Pathogenic
VUS
Benign
Spl.
Splice site mutation
Events
CN-LOH
Dup - LOH
Del - LOH
Grade
I
II
III
NA
PAM50
Luminal A
Luminal B
TNBC
NA

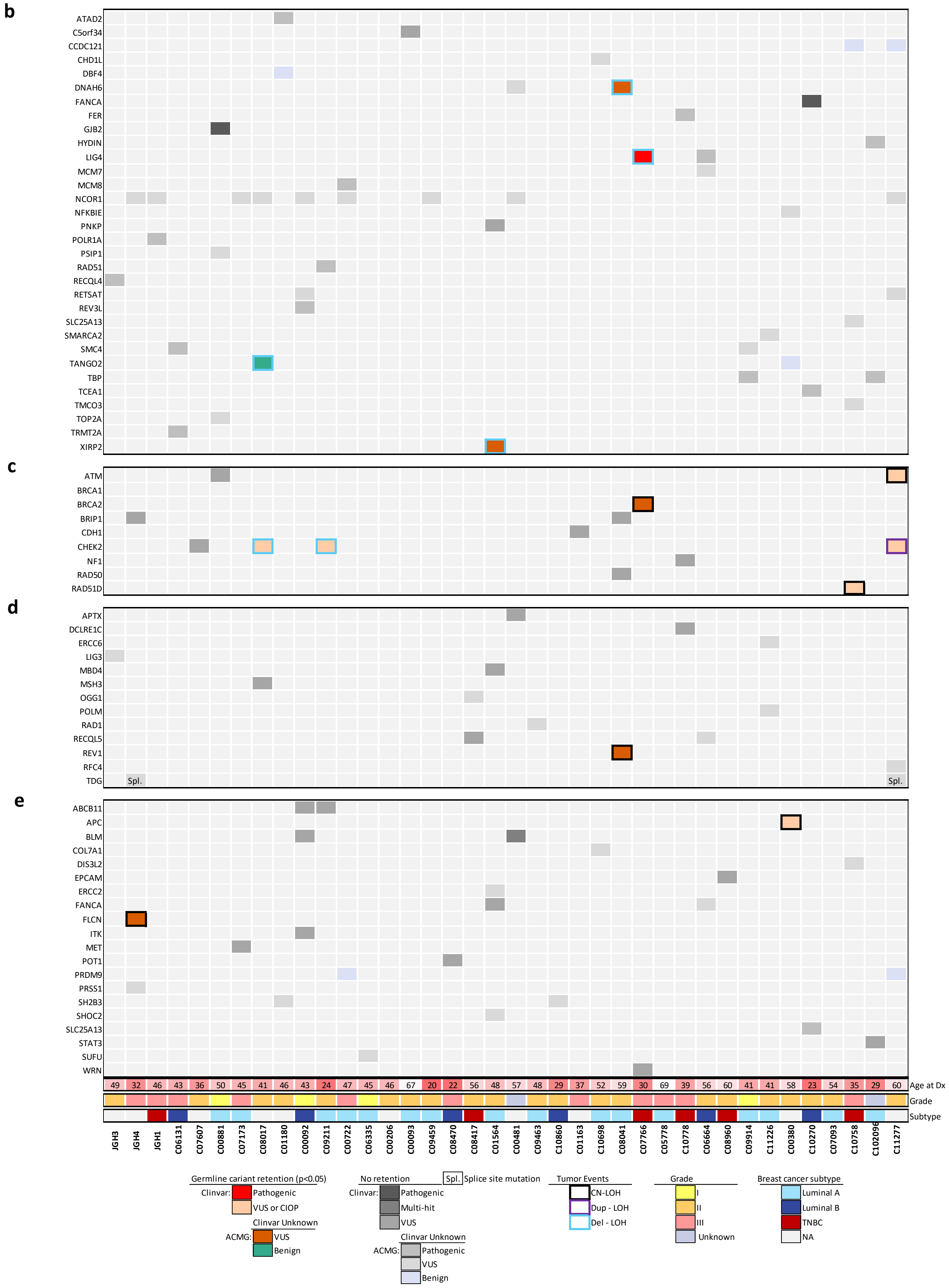
b
ATAD2
C5orf34
CCDC121
CHD1L
DBF4
DNAH6
FANCA
FER
GJB2
HYDIN
LIG4
MCM7
MCM8
NCOR1
NFKBIE
PNKP
POLR1A
PSIP1
RAD51
RECQL4
RETSAT
REV3L
SLC25A13
SMARCA2
SMC4
TANGO2
TBP
TCEA1
TMCO3
TOP2A
TRMT2A
XIRP2
c
ATM
BRCA1
BRCA2
BRIP1
CDH1
CHEK2
NF1
RAD50
RAD51D
d
APTX
DCLRE1C
ERCC6
LIG3
MBD4
MSH3
OGG1
POLM
RAD1
RECQL5
REV1
RFC4
TDG
Spl.
Spl.
e
ABCB11
APC
BLM
COL7A1
DIS3L2
EPCAM
ERCC2
FANCA
FLCN
ITK
MET
POT1
PRDM9
PRSS1
SH2B3
SHOC2
SLC25A13
STAT3
SUFU
WRN
49 32 46 43 36 50 45 41 46 43 24 47 45 46 67 20 22 56 48 57 48 29 37 52 59 30 69 39 56 60 41 41 58 23 54 35 29 60
Age at Dx
Grade
Subtype
JGH3 JGH4 JGH1 C06131 C07607 C00881 C07173 C08017 C01180 C00092 C09211 C00722 C06335 C00206 C00093 C09459 C08470 C08417 C01564 C00481 C09463 C10860 C01163 C10698 C08041 C07766 C05778 C10778 C06664 C08960 C09914 C11226 C00380 C10270 C07093 C10758 C102096 C11277
Germline cariant retention (p<0.05)
Clinvar: Pathogenic
VUS or CIOP
Clinvar Unknown
ACMG: VUS
Benign
No retention
Clinvar: Pathogenic
Multi-hit
VUS
Clinvar Unknown
ACMG: Pathogenic
VUS
Benign
Spl. Splice site mutation
Tumor Events
CN-LOH
Dup - LOH
Del - LOH
Grade
I
II
III
Unknown
Breast cancer subtype
Luminal A
Luminal B
TNBC
NA

Supplementary Figure 1 | Germline landscape. Comutation plot showing variants found in the 864 genes after applying our algorithm **a.** in C-HRGs missense and splice-site **b.** in all truncating variants (nonsense, and frameshift) **c**. in BCSGs missense and splice-site **d.** in DNA repair genes missense and splice-site **e.** in CSGs missense and splice-site. LOH corresponds to copy neutral LOH, Dup-LOH to LOH associate with a duplication, Del-LOH to a hemizygous deletion.

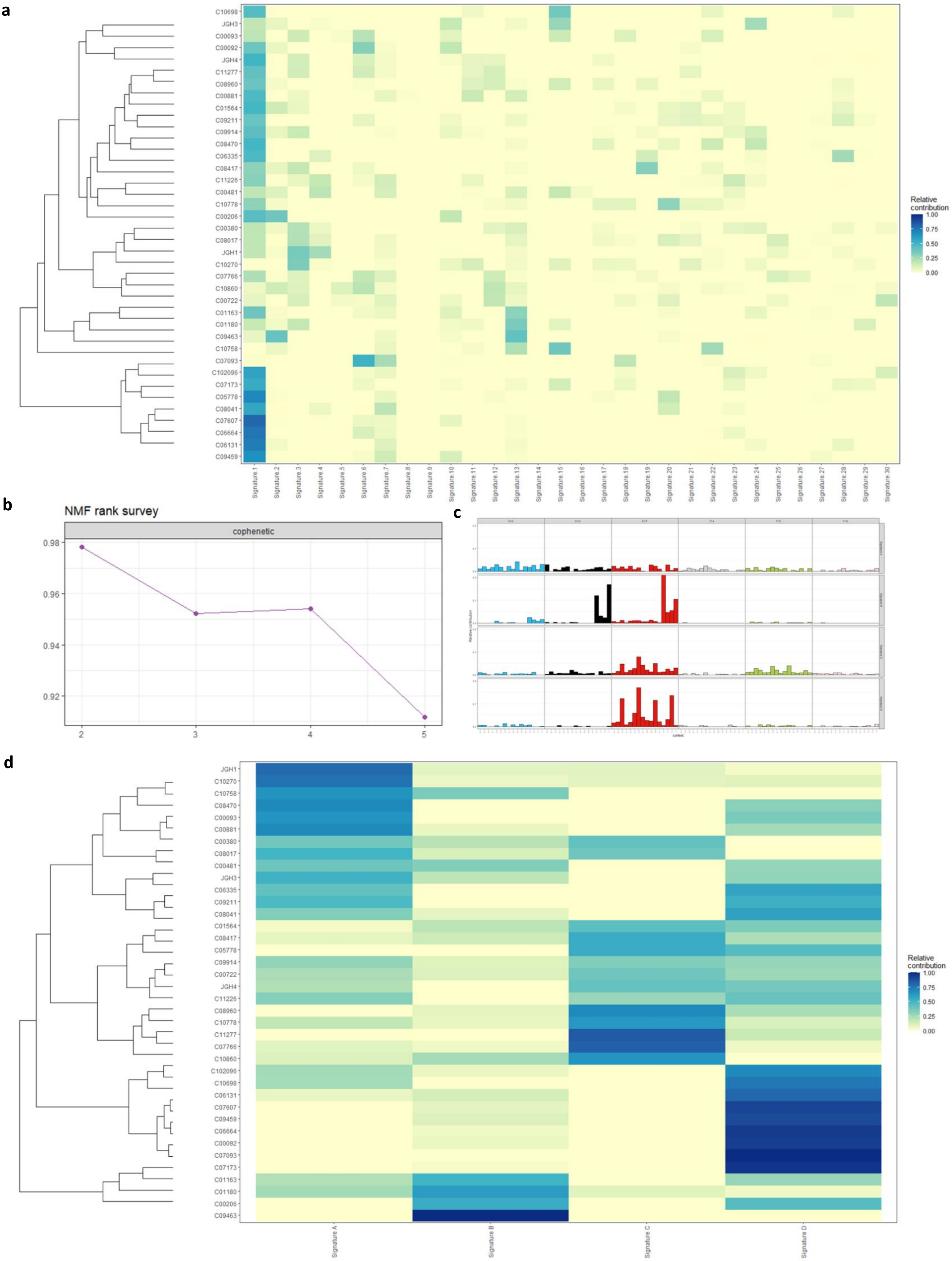
a
C10698
JGH3
C00093
C00092
JGH4
C11277
C08960
C00881
C01564
C09211
C09914
C08470
C06335
C08417
C11226
C00481
C10778
C00206
C00380
C08017
JGH1
C10270
C07766
C10860
C00722
C01163
C01180
C09463
C10758
C07093
C102096
C07173
C05778
C08041
C07607
C06664
C06131
C09459
Signature.1
Signature.2
Signature.3
Signature.4
Signature.5
Signature.6
Signature.7
Signature.8
Signature.9
Signature.10
Signature.11
Signature.12
Signature.13
Signature.14
Signature.15
Signature.16
Signature.17
Signature.18
Signature.19
Signature.20
Signature.21
Signature.22
Signature.23
Signature.24
Signature.25
Signature.26
Signature.27
Signature.28
Signature.29
Signature.30
Relative contribution
1.00
0.75
0.50
0.25
0.00
b
NMF rank survey
cophenetic
0.98
0.96
0.94
0.92
2
3
4
5
c
d
JGH1
C10270
C10758
C08470
C00093
C00881
C00380
C08017
C00481
JGH3
C06335
C09211
C08041
C01564
C08417
C05778
C09914
C00722
JGH4
C11226
C08960
C10778
C11277
C07766
C10860
C102096
C10698
C06131
C07607
C09459
C06664
C00092
C07093
C07173
C01163
C01180
C00206
C09463
Signature A
Signature B
Signature C
Signature D
Relative contribution
1.00
0.75
0.50
0.25
0.00

Supplementary Figure 2 | Mutational Signature landscape. **a.** Heatmap showing the relative contribution of the COSMIC signatures in each sample, with clustering. **b.** NMF ranking determining the minimal number of signatures in the samples by a cophenetic correlation. Here it was 4. **c.** 96-profile of the four minimal signatures. Signature A can be relied on Signature 3, Signature B on the APOBEC signature 3, Signature C on signature 6 or 12, and Signature D on the Aging signature. **d.** Heatmap showing the relative contribution of the four minimal signatures in each sample, with clustering. 9 have a Sig A contribution of more than 50%.

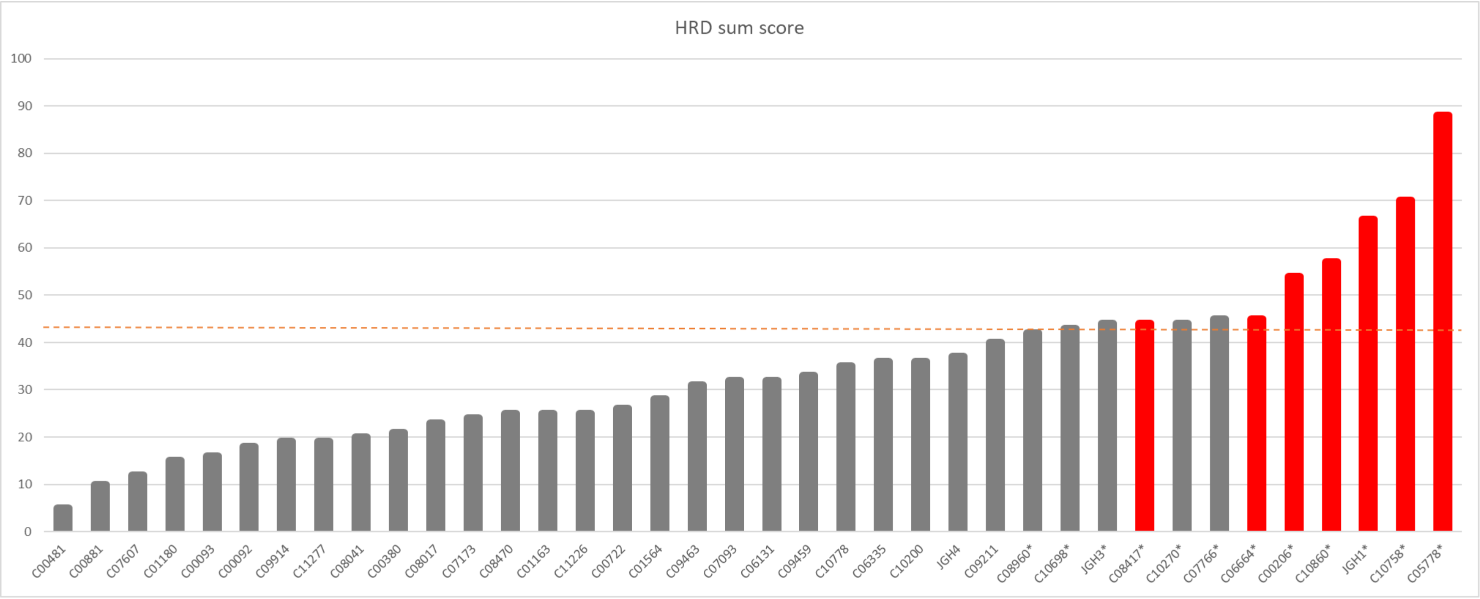

Supplementary Figure 3 | HRD detection using ScarHRD. ScarHRD provides a HRD sum score combining HRD-LOH score, LST score and TAI score. A score ≥ 42 defined HRD status, and tumors classified by ScarHRD are delineated by the orange line. Tumors with score above 41 are marked by *. In red are tumors classified by SigMA as HRD.

a b

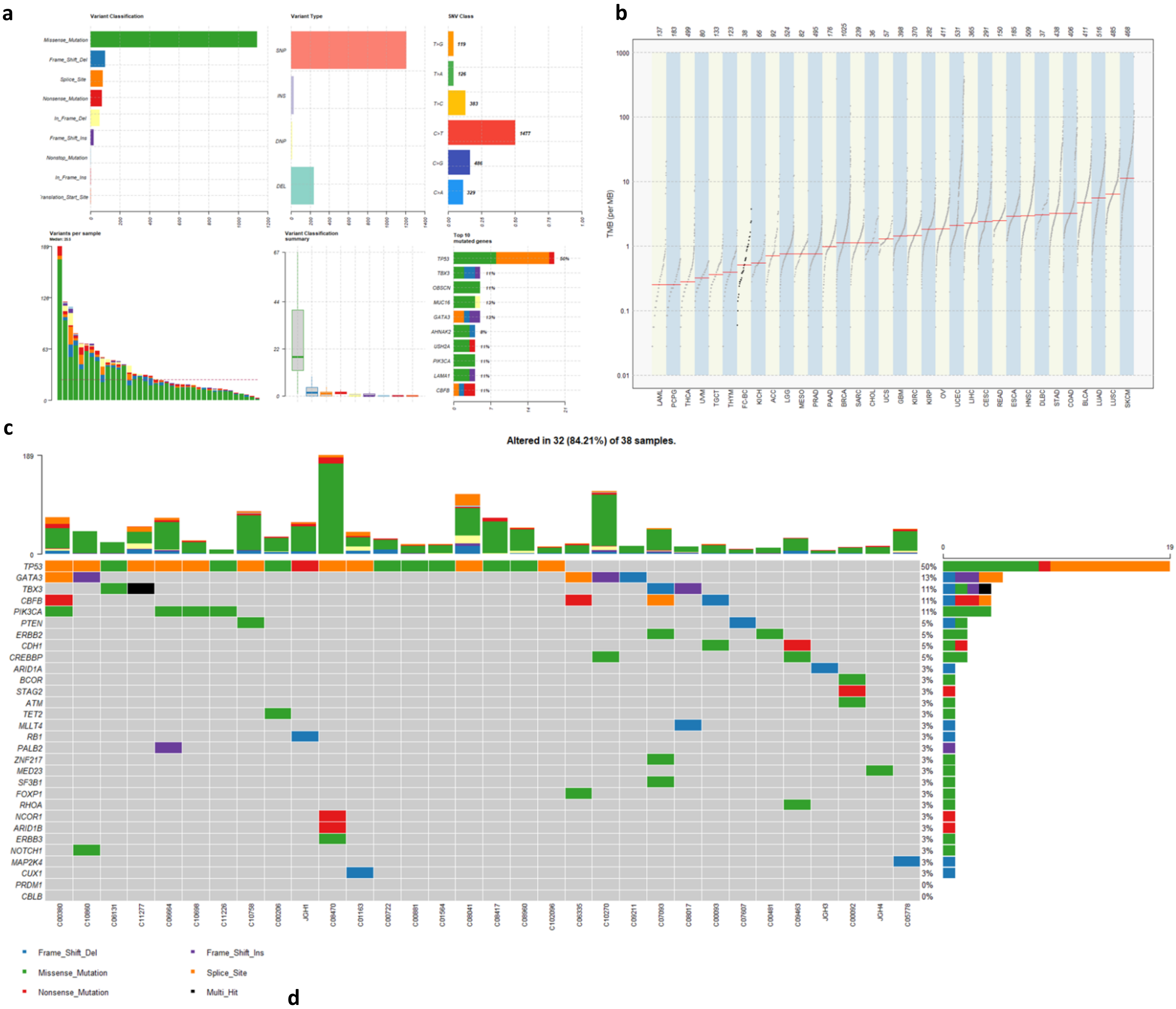

d

q-score	*TP53*	*GATA3*	*CBFB*
MutSigCV	<0.001	<0.01	<0.01
OncodriveFML	0.7	0.9	0.1
MutPanning	<0.001	1.0	0.03
FishHook	<0.001	<0.001	<0.001
	3 / 4	2 / 4	3 / 4

Supplementary Figure 4 | Somatic landscape (SNV). a. Summary of somatic variants found in tumors. **b.** Comparison of mutation burden against 33 TGCA cancer type, this study is called the "FC-BC". **c.** Comutation plot of all breast cancer genes present in the tumors. **d.** Comparison of q-value provides by the driver detection tools. Only genes with at least 1 significant q-value (<0.01) were considered as a driver among breast cancer genes.

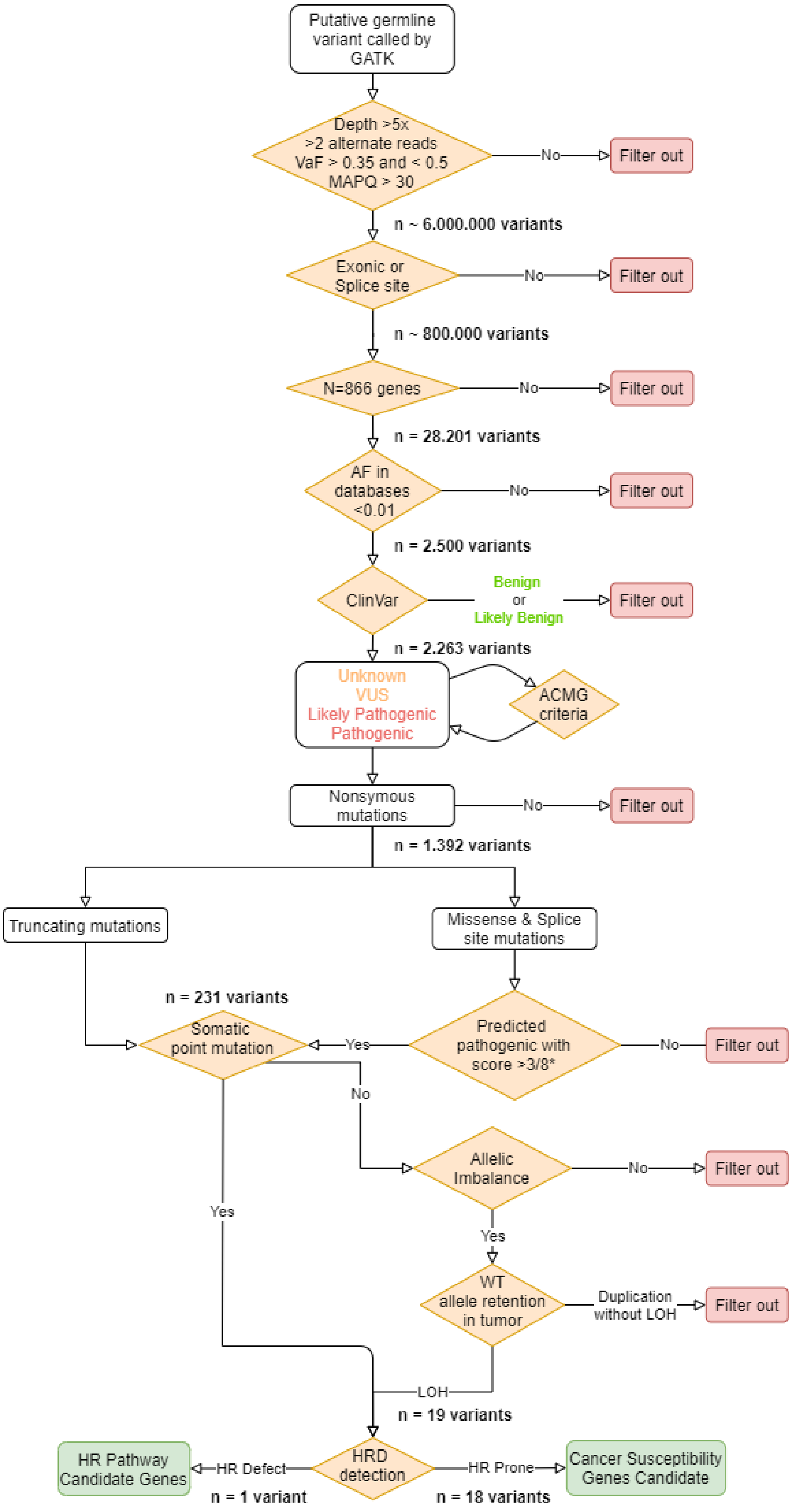

Supplementary Figure 5 | Candidate variant detection algorithm. VaF: Variant allele frequency, MAPQ: Mapping quality Phred score, AF: Allele frequency, WT: Wild-Type. *not for BCSGs
