# Supplementary material for: Current gene panels account for nearly all homologous recombination repair-associated multiple-case breast cancer families
Source: NPJ Breast Cancer. 2021 Aug 25;7:109. doi: 10.1038/s41523-021-00315-8 (PMC8387362; doi:10.1038/s41523-021-00315-8)
Supplement: Supplementary file 3 — Reporting Summary [file 41523_2021_315_MOESM3_ESM.pdf]

nature research

Corresponding author(s): William D Foulkes : william.foulkes@mcgill.ca or Paz Polak : paz.polak@mssm.edut;

Last updated by author(s): Jun 11, 2021

Reporting Summary

Nature Research wishes to improve the reproducibility of the work that we publish. This form provides structure for consistency and transparency in reporting. For further information on Nature Research policies, see our Editorial Policies and the Editorial Policy Checklist.

Statistics

For all statistical analyses, confirm that the following items are present in the figure legend, table legend, main text, or Methods section.

n/a	Confirmed	
☐	☒	The exact sample size (*n*) for each experimental group/condition, given as a discrete number and unit of measurement
☒	☐	A statement on whether measurements were taken from distinct samples or whether the same sample was measured repeatedly
☒	☐	The statistical test(s) used AND whether they are one- or two-sided<br>*Only common tests should be described solely by name; describe more complex techniques in the Methods section.*
☒	☐	A description of all covariates tested
☒	☐	A description of any assumptions or corrections, such as tests of normality and adjustment for multiple comparisons
☒	☐	A full description of the statistical parameters including central tendency (e.g. means) or other basic estimates (e.g. regression coefficient) AND variation (e.g. standard deviation) or associated estimates of uncertainty (e.g. confidence intervals)
☐	☒	For null hypothesis testing, the test statistic (e.g. *F*, *t*, *r*) with confidence intervals, effect sizes, degrees of freedom and *P* value noted<br>*Give P values as exact values whenever suitable.*
☒	☐	For Bayesian analysis, information on the choice of priors and Markov chain Monte Carlo settings
☒	☐	For hierarchical and complex designs, identification of the appropriate level for tests and full reporting of outcomes
☒	☐	Estimates of effect sizes (e.g. Cohen's *d*, Pearson's *r*), indicating how they were calculated

Our web collection on statistics for biologists contains articles on many of the points above.

Software and code

Policy information about availability of computer code

Data collection	This study does not include any new data collection.
Data analysis	For this paper we used either programs from GATK 4.1.6.0, either R packages on R 4.0.3 or either from webtools described bellow:<br>- For call germline and somatic variant we used HaplotypeCaller (https://gatk.broadinstitute.org/hc/en-us/articles/360041415292-HaplotypeCaller) and Mutect2 (https://gatk.broadinstitute.org/hc/en-us/articles/360041416112-Mutect2) from GATK.<br>- For call copy number alteration we followed GATK best practice (https://gatk.broadinstitute.org/hc/en-us/articles/360035531092--How-to-part-I-Sensitively-detect-copy-ratio-alterations-and-allelic-segments and https://gatk.broadinstitute.org/hc/en-us/articles/360035890011--How-to-part-II-Sensitively-detect-copy-ratio-alterations-and-allelic-segments).<br>- Recurrent focal amplifications and deletions in tumors were assess through GISTIC2.0 (https://www.genepattern.org/modules/docs/GISTIC_2.0) on GenePattern webtool.<br>- Variants were annoted with wANNOVAR for germline variant (http://wannovar.wglab.org/index.php) and Ensembl Variant Effect Predictor (VEP) for somatic variant (https://grch37.ensembl.org/info/docs/tools/vep/index.html).<br>- To infer the integer copy number of each pair of alleles as well as LOH, we used cnv_facets on R (https://github.com/dariober/cnv_facets) as well as Sequenza (https://bitbucket.org/sequenzatools/sequenza/src/master/).<br>- To infer drivers of oncogenesis we used MutSigCV (https://www.genepattern.org/modules/docs/MutSigCV) on GenePattern webtool; OncodriveFML from BBG Lab (http://bbglab.irbbarcelona.org/oncodrivefml/home); MutPanning v.2 (https://www.genepattern.org/modules/docs/MutPanning) on GenePattern webtool; and FishHook from Mski Lab on R (https://github.com/mskilab/fishHook).<br>- To assess mutational signature, we used MutationalPatterns from Bioconductor on R (http://www.bioconductor.org/packages/release/bioc/html/MutationalPatterns.html); Maftools from Bioconductor on R (http://www.bioconductor.org/packages/release/bioc/html/maftools.html); and DeconstructSigs on R (https://github.com/raerose01/deconstructSigs).<br>- To assess HRD signature we used SigMA from Park Lab (https://github.com/parklab/SigMA).<br>- To assess HRD genomic scars we used ScarHRD from Sztupinszki Lab (https://github.com/sztup/scarHRD).

- Variants were visualized through Integrative Genomics Viewer (http://igv.org/).

For manuscripts utilizing custom algorithms or software that are central to the research but not yet described in published literature, software must be made available to editors and reviewers. We strongly encourage code deposition in a community repository (e.g. GitHub). See the Nature Research guidelines for submitting code & software for further information.

Data

Policy information about availability of data

All manuscripts must include a data availability statement. This statement should provide the following information, where applicable:

- Accession codes, unique identifiers, or web links for publicly available datasets
- A list of figures that have associated raw data
- A description of any restrictions on data availability

Tumor and germline datasets generated during the current study are not publicly available because the consent provided by study participants did not include a provision for widespread disclosure, but direct requests can be made to the corresponding author for access.

Field-specific reporting

Please select the one below that is the best fit for your research. If you are not sure, read the appropriate sections before making your selection.

☒ Life sciences ☐ Behavioural & social sciences ☐ Ecological, evolutionary & environmental sciences

For a reference copy of the document with all sections, see nature.com/documents/nr-reporting-summary-flat.pdf

Life sciences study design

All studies must disclose on these points even when the disclosure is negative.

Sample size	Samples were obtained from unsolved archived cases, from our clinical genetic department. All were women with breast cancer diagnosed between 1995 and 2017
Data exclusions	We exluded 21 tumors due to either poor quality or insufficient quantity of DNA
Replication	No
Randomization	No
Blinding	No

Reporting for specific materials, systems and methods

We require information from authors about some types of materials, experimental systems and methods used in many studies. Here, indicate whether each material, system or method listed is relevant to your study. If you are not sure if a list item applies to your research, read the appropriate section before selecting a response.

Materials & experimental systems

n/a	Involved in the study	
☒	☐	Antibodies
☒	☐	Eukaryotic cell lines
☒	☐	Palaeontology and archaeology
☒	☐	Animals and other organisms
☒	☐	Human research participants
☐	☒	Clinical data
☒	☐	Dual use research of concern

Methods

n/a	Involved in the study	
☒	☐	ChIP-seq
☒	☐	Flow cytometry
☒	☐	MRI-based neuroimaging

Clinical data

Policy information about clinical studies

All manuscripts should comply with the ICMJE guidelines for publication of clinical research and a completed CONSORT checklist must be included with all submissions.

Clinical trial registration	*Provide the trial registration number from ClinicalTrials.gov or an equivalent agency.*
Study protocol	*Note where the full trial protocol can be accessed OR if not available, explain why.*
Data collection	We used FFPE tumors from women with breast cancer diagnosed between 1995 and 2017, as well as their blood.

Outcomes | *Describe how you pre-defined primary and secondary outcome measures and how you assessed these measures.*
